# Integrated transcriptome and metabolome analysis of salinity tolerance in response to foliar application of choline chloride in rice (*Oryza sativa* L.)

**DOI:** 10.3389/fpls.2024.1440663

**Published:** 2024-08-01

**Authors:** Jingxin Huo, Minglong Yu, Naijie Feng, Dianfeng Zheng, Rui Zhang, Yingbin Xue, Aaqil Khan, Hang Zhou, Wanqi Mei, Xiaole Du, Xuefeng Shen, Liming Zhao, Fengyan Meng

**Affiliations:** ^1^ College of Coastal Agricultural Sciences, Guangdong Ocean University, Zhanjiang, China; ^2^ South China Branch of National Saline-Alkali Tolerant Rice Technology Innovation Center, Zhanjiang, China; ^3^ Shenzhen Institute of Guangdong Ocean University, Shenzhen, China

**Keywords:** salt tolerance, rice, photosynthesis, transcriptome, metabolome, carbon metabolism

## Abstract

**Introduction:**

Salt stress is a major abiotic stress that affects crop growth and productivity. Choline Chloride (CC) has been shown to enhance salt tolerance in various crops, but the underlying molecular mechanisms in rice remain unclear.

**Methods:**

To investigate the regulatory mechanism of CC-mediated salt tolerance in rice, we conducted morpho-physiological, metabolomic, and transcriptomic analyses on two rice varieties (WSY, salt-tolerant, and HHZ, salt-sensitive) treated with 500 mg·L^-1^ CC under 0.3% NaCl stress.

**Results:**

Our results showed that foliar application of CC improved morpho-physiological parameters such as root traits, seedling height, seedling strength index, seedling fullness, leaf area, photosynthetic parameters, photosynthetic pigments, starch, and fructose content under salt stress, while decreasing soluble sugar, sucrose, and sucrose phosphate synthase levels. Transcriptomic analysis revealed that CC regulation combined with salt treatment induced changes in the expression of genes related to starch and sucrose metabolism, the citric acid cycle, carbon sequestration in photosynthetic organs, carbon metabolism, and photosynthetic antenna proteins in both rice varieties. Metabolomic analysis further supported these findings, indicating that photosynthesis, carbon metabolism, and carbon fixation pathways were crucial in CC-mediated salt tolerance.

**Discussion:**

The combined transcriptomic and metabolomic data suggest that CC treatment enhances rice salt tolerance by activating distinct transcriptional cascades and phytohormone signaling, along with multiple antioxidants and unique metabolic pathways. These findings provide a basis for further understanding the mechanisms of metabolite synthesis and gene regulation induced by CC in rice in response to salt stress, and may inform strategies for improving crop resilience to salt stress.

## Highlights

- The exogenous choline chloride treatment can significantly induce gene expression, physiological changes, and metabolism related to photosynthesis, thereby alleviating the impact of salt stress in rice.

## 1 Introduction

Rice (*Oryza sativa* L.) is the second most widely cultivated crops worldwide as the staple food for more than half of the global population, especially in Asian-Pacific region (Mainuddin et al., 2021; Yuan et al., 2021). Soil salinity, a major environmental stress that restricts crop growth and productivity (Zhao et al., 2020). Rising salinity in the agricultural field considerably affects crop productivity and plant development (Chauhan et al., 2022). Globally the most detrimental and observable impact of salt stress on rice plants is a reduction of the shoot and root growth, plant biomass and ultimately a decrease in grain yield production (Das et al., 2015). Compared to other crops like wheat and barley, rice is extremely susceptible to salt stress (Ganie et al., 2019) and more than 50% yield losses with soil electrical conductivity of 6 dS/m (Al-Tamimi et al., 2021). The application of salt-tolerant plants and the improvement of plant salt tolerance are recognized as the major routes for saline soil restoration and utilization (Wang et al., 2022).

Foliar application of chemical regulators to enable plants to tolerate abiotic stress has become an effective and practical strategy (Du et al., 2023; Li et al., 2023; Zhou et al., 2023). According to Council Directive 70/524/EEC8, Choline chloride is a cheap, biodegradable, and non-toxic salt, which acts as a nutritional additive for all species without time limitation (Additives and Products or Substances used in Animal, 2011). CC, an organic compound, is the hydrochloride salt of choline with the formula C_5_H_14_ClNO, and it is frequently used as a dietary additive (Yara et al., 2015). Choline is the precursor of phosphatidylcholine, effectively ensuring protection for membrane fluidity and permeability in the membrane transport system (Calderon et al., 2013). CC is an efficient reactive oxygen species (ROS) scavenger that can mitigate lipid peroxidation and protect membranes (Singh and Satheeshkumar, 2024). Moreover, CC can improve plant growth and development (Mcneil et al., 2001). CC application enhanced the tolerance of cluster bean (*Cyamopsis tetragonoloba* L.) against salinity stress (Riaz et al., 2021).

Photosynthesis converts solar energy into chemical energy stored in plants (Gao et al., 2022). Photosynthesis is crucial biochemical process of energy production in higher plants and is very sensitive to salt stress (Meloni et al., 2003). The carbon metabolic pathway enables the conversion between starch and sugar (Li et al., 2024). Salt stress disrupts chloroplast structure and degrade chlorophyll content which block carbon metabolism pathway (Yan et al., 2021b). When carbohydrates synthesized by light and carbon fixation cannot meet catabolic needs under abiotic stress, plants redistribute the limited carbohydrates in the organ to improve utilization efficiency, thus ensuring growth and development under unfavorable conditions. Therefore, it is mandatory to enhance overall photosynthesis and crop productivity under saline soils for the survival of all living biota.

As a photosynthesis promoter, CC is vital for increasing grain yield (Yan et al., 2020).It is unclear whether CC can mitigate stress damage under salt stress by promoting photosynthesis and altering carbohydrate conversion pathways. Therefore, in-depth study of the response mechanism of rice to salt stress after CC application laid a foundation for understanding the resistance mechanism of CC-induced plants. The current study was intended: (A) To identify whether CC can alleviate the effects of salt stress on rice seedlings and contribute to their adaptation before transplanting to saline-alkali land. (B) Investigate the effects of CC on key enzyme activities involved in photosynthetic process and carbon metabolism of rice seedlings under salt stress. (C) To understand the pathways of CC in alleviating salt stress of rice seedlings from the gene expression level and metabolic level through transcriptomics and metabolomics approaches. It provides a new perspective and scientific basis for understanding the adaptation mechanism of rice to salt stress. The results provide new insights into the regulation of salt tolerance in rice and have important significance for agricultural production and plant stress biology research.

## 2 Materials and methods

### 2.1 Plant material and growth conditions

The experiment was conducted in the solar greenhouse of national saline-tolerant rice technology innovation center, Zhanjiang, Guangdong Province, China. The salt-sensitive indica rice HuangHuaZhan (HHZ) and salt-tolerant hybrid rice Wanshengyou-TianhongNo.4 (WSY) were used as experimental materials. Rice seeds were disinfected with 3% H_2_O_2_ for 13 min, washed thoroughly with distilled water, and soaked in water at 30°C for 24 h to accelerate germination. Then, seeds with the same dew white were selected and seeded in a nutrient bowl of 8.5×6.7×9.1cm, with plant spacing of 1cm, and regular water management was performed until the V3 growth stage. The soil used in the pot is a 3:1 volume mixture of red loam and sand.

The treatment time and sampling time in the experiment were mainly based on the growth stage of rice seedlings. Rice seedlings of two varieties growing to three-leaf single-minded phase (V3, 23 days after seeding) were randomly divided into 4 groups and treated separately: irrigated with water and sprayed with distilled water (CK), irrigated water and sprayed with 500 mg·L^-1^ Choline Chloride (CC), irrigated with 0.3% NaCl solution and sprayed with distilled water (S), irrigated with 0.3% NaCl solution and sprayed with 500 mg·L^-1^ Choline Chloride (SCC).

In each group, the same amount of distilled water or CC was sprayed once only in the V3 growth stage, and the same amount (about 2 mL) of solution was obtained by spraying evenly on the front and back sides of each leaf with the help of a manual sprayer. After that, the salt solution or water with a predetermined concentration was irrigated regularly and quantitatively every other day. The irrigation volume was 3 times the water capacity, and about 2/3 of the solution flowed out so that the remaining salt was washed away to keep the salt concentration constant. The same amount of water was irrigated in the control group. Salt stress was maintained after 48 hours of the spraying interval until sampling was completed. After 24 hours of salt stress, the middle part of the penultimate leaf of the plant was taken for transcriptomics, metabolomics and qRT-PCR. Salt stress was determined by four-leaf single-phase sampling for morphological and physiological indexes (V4, 30 days after seeding). Phenotypic indices and biomass analysis.

The aboveground portion of rice was used to determine plant height, stem diameter, and leaf area. Roots were scanned with a Win RHIZO LA6400XL root scanner and analyzed with Win RHIZO Pro software to determine root length, surface area, volume, and tip number. The rice seedlings were rinsed and incubated at 105°C for 30 min., dried at 70°C to constant weight, and the aboveground and root dry weights were measured. The aboveground phenotypes and dry weights of 15 biological replicates were determined for each treatment. Root parameters of 8 biological replicates per treatment were also measured.

### 2.2 Determination of gas exchange parameters and photosynthetic pigments

Transpiration rate (*T_r_*), Photosynthetic rate (*P_n_*), Intercellular carbon dioxide concentration (*C_i_*), and Stomatal conductance (*G_s_*) of functional rice leaves were measured using the LI-6800 portable photosynthetic system (LI-COR, USA) from 9:00 am to 11:30 am. During measurement, rice leaves are sandwiched between the leaf chambers of the photosynthetic apparatus, with the light intensity set at 1000 µmol·m^-2^·s^-1^, the CO_2_ concentration controlled at 400 µmol·mol^-1^, the leaf temperature at 25°C, and the relative humidity at 65%. During measurement, insert the blade for 3 min and record the data of gas exchange parameters (Meng et al., 2023). Water use efficiency (WUE) was calculated using the formula: WUE = *P_n/_T_r_* (Pathare et al., 2023). Repeat the measurement four times for each process.

The concentrations of chlorophyll a (Chl-a), chlorophyll b (Chl-b), and carotenoid (Car) at 665, 649, and 470 nm were determined by spectrophotometry after 0.02 g fresh leaves were soaked in 1.8 ml 95% ethanol for 24 h. The Chl-a, Chl-b, Car, and total chlorophyll (Chl-a + Chl-b) contents were calculated according to Zuo et al (Zuo et al., 2024).

### 2.3 Physiological and biochemical indices analysis

The contents of fructose, sucrose, soluble sugar, and starch were determined by the acid hydrolysis colorimetric method, anthrone-sulfuric acid method, and 3,5-dinitrosalicylic acid colorimetric method as described by Kuai et al (Kuai et al., 2014). According to the method of Singh et al (Singh et al., 2021), the activities of sucrose synthetase (SS) and sucrose phosphate synthetase (SPS) were determined by resorcinol colorimetry. The activities of α-amylase, β-amylase, and total amylase were determined by the colorimetric method of Dai et al (Dai et al., 2016).

### 2.4 RNA sequencing and transcriptomics analysis

The total RNA from 24 samples was extracted using CTAB-PBIOZOL reagent according to the standard protocol. Qualitative and quantitative analysis of total RNA was performed using a Nano Drop and Agilent 2100 biological analyzer (Thermo Fisher Scientific, MA, USA). The cDNA library was then constructed and sequenced on the BGISEQ platform, and the length of the generated reads was 150 bp paired-end (PE 150). To obtain clear reads, the low-quality reads, adapter sequences and sequences with more than 10% poly-N in the raw reads were filtered using fastp 0.21.0. The Q20, Q30, and GC content of each sample were calculated. Clear reads from each sample were mapped to the Nipponbare (*Oryza sativa ssp.* Japonica) reference genome using HISAT to align the clean reads to the reference genome and using Bowtie2 to align the clean reads to the reference genes (GCF_001433935.1_IRGSP-1.0). Raw counts of genes were performed using feature counts. The differentially expressed genes (DEGs) between two samples were identified using DESeq2 with the parameters of |log_2_fold change| ≥ 1 and false discovery rate (FDR) < 0.05. The hyper-geometric distributions of Kyoto Encyclopedia of Genes and Genomes (KEGG) pathways^1^ was tested.

### 2.5 Sample preparation and extraction for widely targeted metabolomics

Metabolite profiles were determined using the same materials as transcriptome analysis. The extract analysis, metabolite identification, and quantification were performed by BGI (China) following their standard procedures. After homogenizing at 50 Hz for 5 min using Tissue Lyser (JXFSTPRP, China), samples were sonicated for 30 min at 4°C and incubated at -20°C for 1 hour. The samples were further centrifuged for 15 min at 14,000 rpm, 4°C. 600 μL of the supernatants were filtered through 0.22 μ m microfilters and transferred to autosampler vials for LC-MS analysis. A quality control (QC) sample was prepared by pooling 20 μL supernatant of each sample to evaluate the reproducibility and stability of the whole LC-MS analysis. The sample analysis was performed on a Waters ACQUITY UPLC 2D (Waters, USA), coupled to a Q-Exactive mass spectrometer (Thermo Fisher Scientific, USA) with a heated electrospray ionization (HESI) source. Differential metabolites screening criteria: (1) the VIP values of the first two PCs of the PLS-DA model ≥1, (2) Fold-Change≥1.2 or ≤0.83, (3) q-value<0.05.

### 2.6 Confirmation of gene expression patterns by qRT-PCR

A set of 12 DEGs was selected to confirm the expression level of microarray results using real-time-PCR (RT-PCR). The sequences corresponding to the genes were obtained from the rice genome sequences database (TIGR), and the sequences of exons of each gene were used to design the RT-PCR primers using Primer6.0 software (**Supplementary Table 1**); the *Actin* (Phule et al., 2018) and *UBQ5* (Auler et al., 2017) were used as internal reference genes. As described previously, the same RNA samples for the transcriptomics analysis were used for RT-PCR. The SYBR Green Premix Pro Taq HS qPCR Kit (Novizan Biotechnology Co., LTD, Nanjing, China) was used for the RT-qPCR assay using 20 μL reaction solutions containing that 10 μL 2 × SYBR real-time PCR premixture, 0.4 μL primer F, 0.4 μL primer R, 1 μL cDNA and RNase free dH_2_O up to 20 μL. The qPCR reactions involved denaturation at 95°C for 5 min followed by 40 cycles of 15 s at 95°C and 30 s at 60°C. The RT-qPCR assays were carried out using a QuantStudio Real-Time PCR system (Applied Biosystems, Foster City, CA, USA). The qPCR data were analyzed using the 2^−ΔΔCt^ quantitative method to determine differences in gene expression (Livak and Schmittgen, 2001). Three independent biological replicates and three technological replicates were used for each sample in this study.

## 3 Results

### 3.1 Effects of CC spray on rice seedling phenotype under salt stress

**Figure 1:**
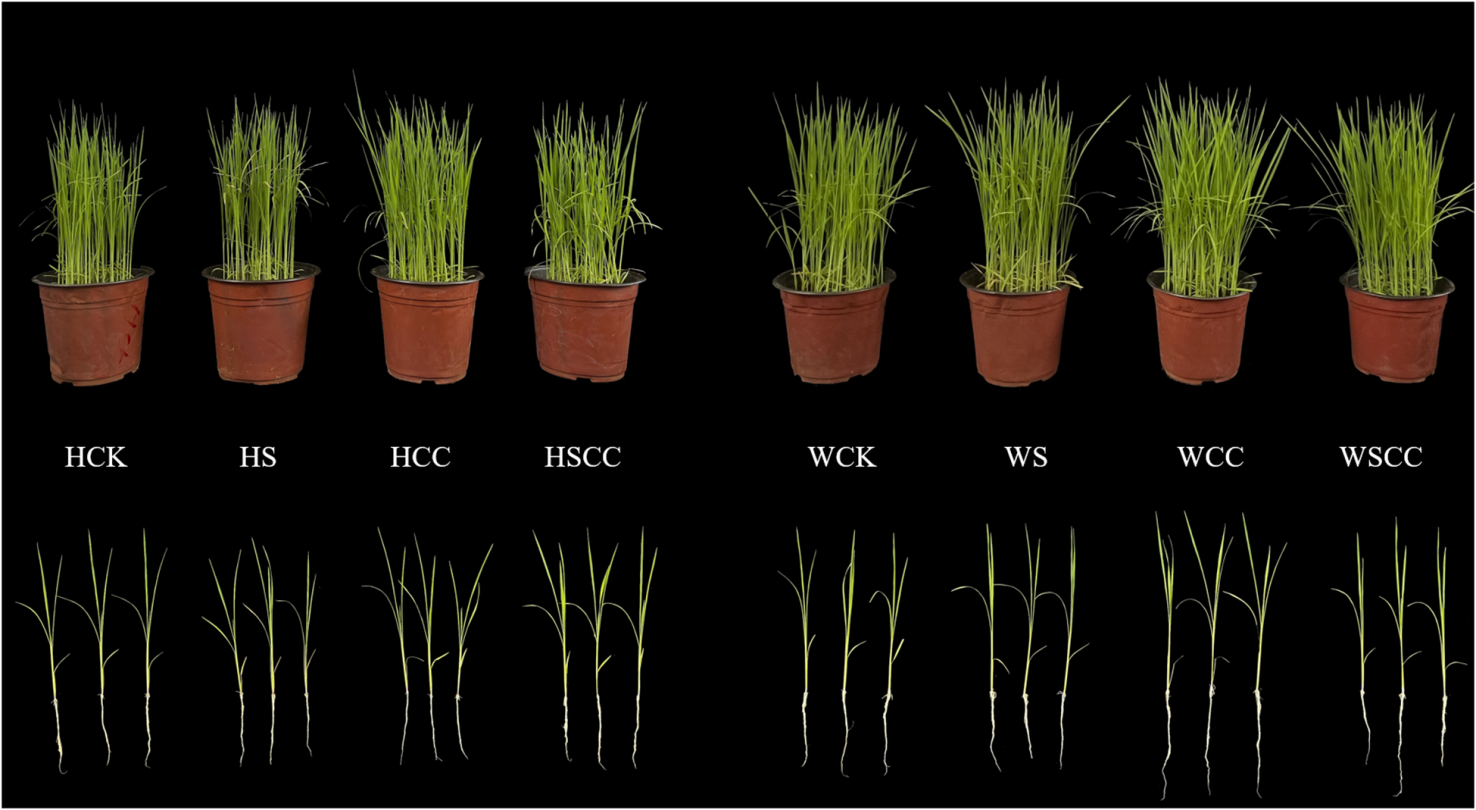
Effects of CC on the growth of rice seedlings under NaCl stress in HHZ and WSY. CK: irrigated water + sprayed water, S: irrigated 0.3% NaCl solution + sprayed water, CC: irrigated water + sprayed water 500 mg·L^-1^ CC, SCC: irrigated 0.3% NaCl solution + sprayed water 500 mg·L^-1^ CC.

**Figure 2:**
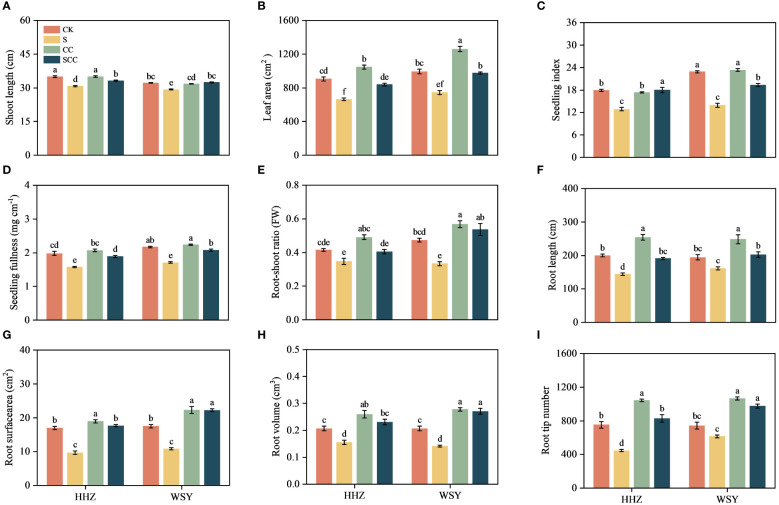
Impact of CC treatment on phenotypes under salt stress. **(A)** Shoot length. **(B)** Leaf area. **(C)** Seeding index. **(D)** Seeding fullness. **(E)** Root-shoot ratio. **(F)** Root length. **(G)** Root surface area. **(H)** Root volume. **(I)** Root tip number. Control (CK), Choline Chloride (CC), Salt (S), Salt and Choline Chloride (SCC). The phenotype data represent the mean of 15 replicates ± SEM. The root phenotype data represent the mean of 8 replicates ± SEM. Different letters indicate the importance of all treatments involving both rice varieties (*p < 0.05*) according to Tukey HSD test.

Rice seedlings illustrated different responses against the three concentration of Choline chloride (CC) (**Figure 1**). Salt stress markedly influenced the seedlings morphological traits of both varieties. Compared with NaCl treatment, the combine treatment (SCC) significantly alleviated the inhibitory effects of salt stress (**Figure 2**).

The salt stress significantly decreased the shoot length by 11.74% and 7.81%, respectively for HHZ and WSY rice varieties. Compared to NaCl treatment, the CC treatment significantly reduced the salt stress (**Figure 2A**). Compared with the CK, NaCl treatment significantly decreased the leaf area by 26.58% and 10.59%, respectively for HHZ and WSY rice varieties. While CC treatment significantly increased the leaf area by 15.65% and 26.98%, respectively for HHZ and WSY rice varieties. Compared to NaCl treatment, the CC treatment significantly enhanced the leaf area by 26.32% and 23.56%, respectively for HHZ and WSY under salt stress (**Figure 2B**). Salt stress markedly decreased seedling index decreased by 28.14% and 39.12%, respectively for HHZ and WSY. Compared with NaCl, CC treatment significantly increased the seeding index by 28.39% and 27.99%, respectively for HHZ and WSY rice varieties (**Figure 2C**). Compared with the CK, the seedling fullness significantly decreased by 20.32% and 21.28%, respectively for HHZ and WSY rice varieties under salt stress. Compared with NaCl, SCC treatment significantly improved the seedling fullness by 19.69% and 21.45%, respectively for HHZ and WSY under salt stress (**Figure 2D**).

Salt stress decreased the by 16.27% and 29.43%, respectively for HHZ and WSY. Compared with NaCl, SCC treatment increased the root shoot ratio of rice seedlings by 16.58% and 60.82%, respectively for HHZ and WSY under salt stress (**Figure 2E**). NaCl treatment reduced root length by 28.06% and 16.87%, root surface area by 43.01% and 38.49%, root volume by 24.84% and 31.54% and root tip number by 40.61% and 17.26%, respectively for HHZ and WSY. Compared with NaCl, the SCC significantly increased root length by 24.81% and 20.23%, root surface area by 45.32% and 51.41%, root volume by 32.27% and 47.63%, and root tip number by 46.01% and 36.99%, respectively for HHZ and WSY (**Figures 2F–I**).

### 3.2 Effect of CC on the photosynthetic pigment content in rice under salt stress

**Figure 3:**
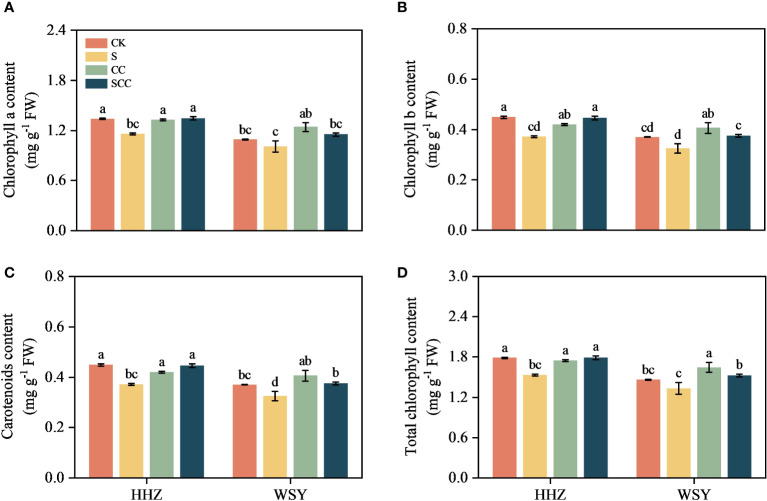
Effect of CC on photosynthetic pigment content in HHZ and WSY under salt stress. **(A)** Chlorophyll a content. **(B)** Chlorophyll b content. **(C)** Carotenoids content. **(D)** Total chlorophyll. Control (CK), Choline Chloride (CC), Salt (S), Salt and Choline Chloride (SCC). Values are the mean ± SD of four replicate samples. Different letters indicate the importance of all treatments involving both rice varieties (*p < 0.05*) according to Tukey HSD test.

Salt stress significantly inhibited chlorophyll synthesis in both rice leaves (**Figure 3**). Compare to CK, NaCl reduced chlorophyll-a by 13.51% and 7.66%, chlorophyll-b by 17.08% and 12.44%, total chlorophyll content by 14.40% and 8.87%, and carotenoids by 14.29% and 9.41%, respectively for HHZ and WSY under salt stress. Similarly in contrast to S, the SCC treatment enhanced chlorophyll-a by 13.91% and 12.37%, chlorophyll-b by 16.46% and 13.44%, total chlorophyll content by 14.54% and 12.63%, and carotenoids by 14.79% and 13.14%, respectively for HHZ and WSY under salt stress.

### 3.3 Effect of CC on the gas exchange parameters in rice under salt stress

**Figure 4:**
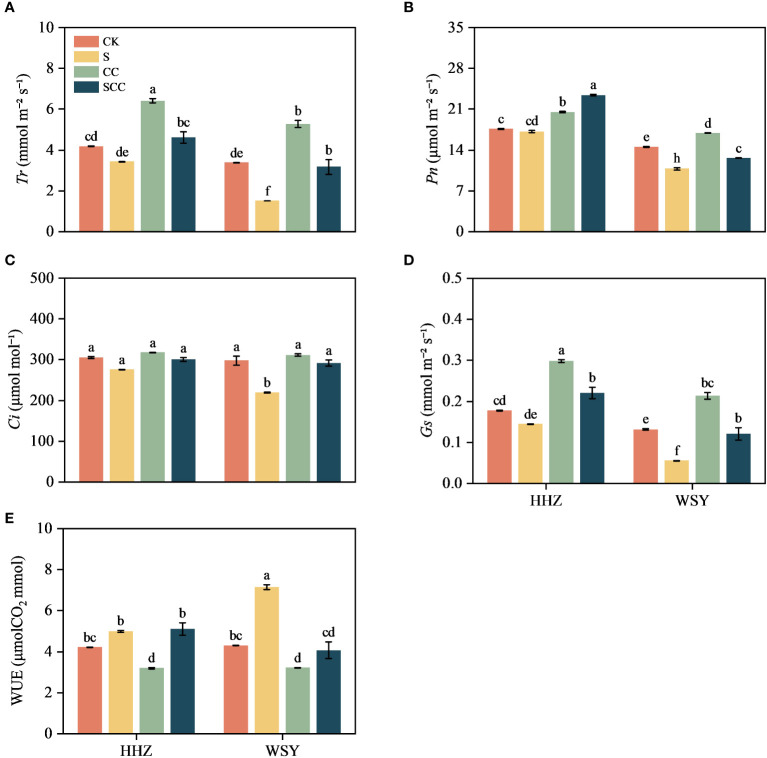
Effect of CC on gas exchange parameters in HHZ and WSY under salt stress. **(A)** Transpiration rate (*T*_r_). **(B)** Net photosynthetic rate (*P*_n_). **(C)** Intercellular CO2 concentration (*C*_i_). **(D)** Stomatal conductance (*G*_s_). **(E)** Water Use Efficiency (WUE). Control (CK), Choline Chloride (CC), Salt (S), Salt and Choline Chloride (SCC). Values are the mean ± SD of four replicate samples. Different letters indicate the importance of all treatments involving both rice varieties (*p < 0.05*) according to Tukey HSD test.

NaCl stress decreased the transpiration rate (*T_r_*), photosynthesis rate (*P_n_*), internal CO_2_ concentration (*C_i_*), stomatal conductance (*G_s_*) values, and improved water use efficiency (WUE) in both varieties (**Figure 4**). In comparison to CK, NaCl treatment decreased by 17.92% and 55.43% in *T_r_*, 2.79% and 25.82% in *P_n_*, 9.61% and 26.38% in *C_i_*, and 18.57% and 57.74% in *G_s_*, respectively for varieties HHZ and WSY under salt stress. However, WUE increased by 18.43% and 66.41%, respectively for HHZ and WSY under salt stress. Compared to S, the SCC treatment significantly reduced the WUE (43.10%) of WSY. Foliar application of exogenous CC significantly alleviated the inhibitory effect of NaCl stress on photosynthetic traits in both rice varieties. Compared to S treatment, SCC treatment increased *T_r_* by 25.58% and 52.50%, *P_n_* by 26.71% and 14.65%, *C_i_* by 8.23% and 24.78%, *G_s_* by 34.32% and 53.75%, respectively for HHZ and WSY under salt stress.

### 3.4 Effect of CC on the carbohydrate content in rice under salt stress

**Figure 5:**
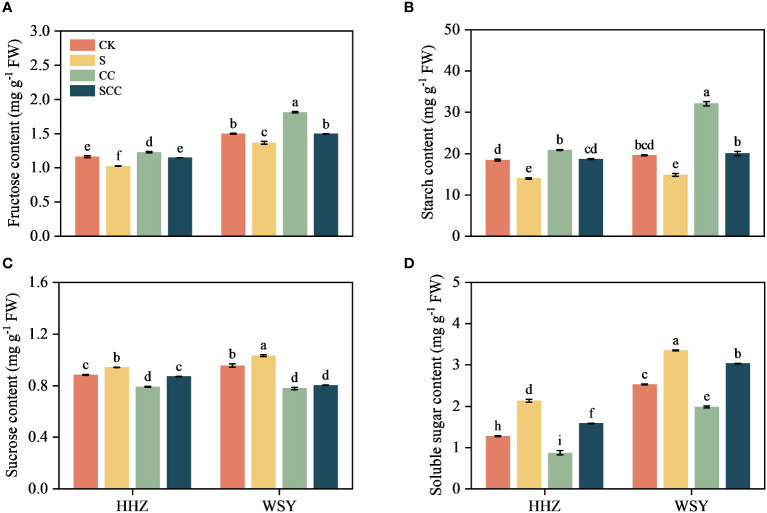
Effect of CC on the carbohydrate content in HHZ and WSY under salt stress. **(A)** Fructose content. **(B)** Starch content. **(C)** Sucrose content. **(D)** Soluble sugar content. Control (CK), Choline Chloride (CC), Salt (S), Salt and Choline Chloride (SCC). Values are the mean ± SD of four replicate samples. Different letters indicate the importance of all treatments involving both rice varieties (*p < 0.05*) according to Tukey HSD test.

Different carbohydrate components of rice leaves showed different trends under salt stress (**Figure 5**). Compared to CK, S treatment significantly decreased fructose content by 11.71% and 8.70%, and starch content by 24.02% and 24.43%, respectively for HHZ and WSY under salt stress. Similarly compared to CK, S treatment significantly increased sucrose content by 6.77% and 8.70% and soluble sugar content by 6.79% and 32.51%, respectively for HHZ and WSY under salt stress. Compared with NaCl, SCC significantly increased fructose content by 10.60% and 8.68% and starch content by 25.08% and 25.82%, respectively for HHZ and WSY under salt stress. Similarly compared with NaCl, SCC significantly increased sucrose content by 7.53% and 22.25% and soluble sugar content by 25.65% and 9.47%, respectively for HHZ and WSY under salt stress.

### 3.5 Effect of CC on sucrose metabolism-related enzymes in rice under salt stress

**Figure 6:**
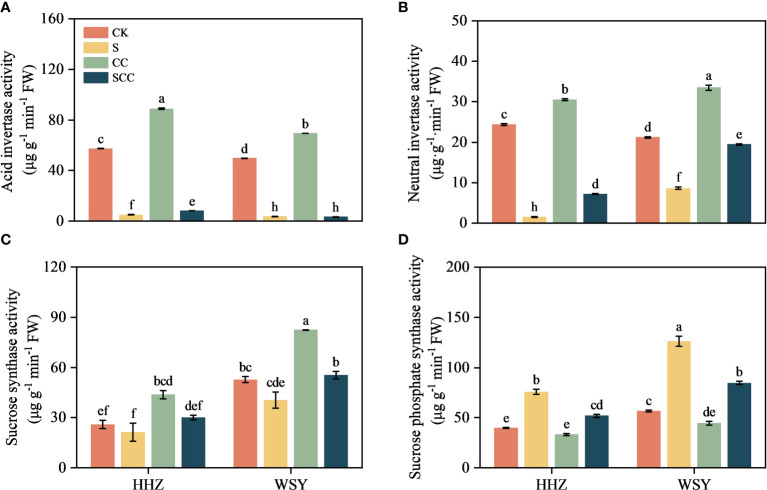
Effect of CC on sucrose metabolism-related enzymes in HHZ and WSY under salt stress. **(A)** Acid invertase activity (AI). **(B)** Neutral invertase activity (NI). **(C)** Sucrose synthase activity (SS). **(D)** Sucrose phosphate synthase activity (SPS). Control (CK), Choline Chloride (CC), Salt (S), Salt and Choline Chloride (SCC). Values are the mean ± SD of four replicate samples. Different letters indicate the importance of all treatments involving both rice varieties (*p < 0.05*) according to Tukey HSD test.

NaCl stress significantly reduced the activities of acid invertase (AI), neutral invertase (NI), and sucrose synthetase (SS) (**Figure 6**). Compared to CK, S treatment decreased AI activity by 91.67% and 92.67%, NI activity by 93.76% and 59.42%, SS activity by 17.88% and 23.25%, respectively for HHZ and WSY under salt stress. Similarly compared to CK, S treatment significantly increased SPS activity by 23.25% and 123.75%, respectively for HHZ and WSY under salt stress. Compared to NaCl, SCC increased NI activity by 78.88% and 55.83%, SS activity by 29.39% and 26.89%, respectively for HHZ and WSY but significantly increased AI activity by 39.42% in HHZ under salt stress. Similarly compared to NaCl, SCC significantly decreased SPS activity by 31.49% and 33.10%, respectively for HHZ and WSY under salt stress.

### 3.6 Effect of CC on starch metabolism-related enzymes in rice under salt stress

**Figure 7:**
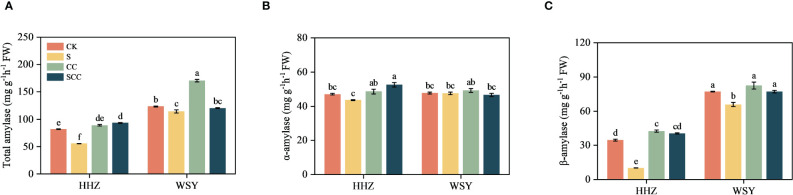
Effect of CC on starch metabolism related enzymes in HHZ and WSY under salt stress. **(A)** Total amylase activity. **(B)** α-amylase activity. **(C)** β-amylase activity. Control (CK), Choline Chloride (CC), Salt (S), Salt and Choline Chloride (SCC). Values are the mean ± SD of four replicate samples. Different letters indicate the importance of all treatments involving both rice varieties (*p < 0.05*) according to Tukey HSD test.

Compared to CK, S treatment significantly decreased total amylase activity by 32.07%, α-amylase activity by 7.3%, β-amylase activity by 70.85% in HHZ under salt stress. Compared to NaCl, SCC significantly increased total amylase activity by 40.45%, α-amylase activity by 16.96%, β-amylase activity by 74.96% in HHZ under salt stress. Rice variety WSY also showed a similar trend to HHZ, but statistically non-significant (**Figure 7**).

### 3.7 RNA-seq analysis after choline chloride treatment to rice seedlings under salt stress

To explore the effect of exogenous CC on the global transcriptome of improving salt tolerance in rice seedlings, RNA sequencing was performed using the leaf of rice seedlings, with three biological replicates for each group (24 samples in total), using BGISEQ platform, averagely generating about 6.63G Gb bases per sample. The average mapping ratio with reference genome is 90.20%, the average mapping ratio with gene is 77.44%, and 25651 genes were identified (**Supplementary Figure 1**).

The average data ratio of sequencing quality to Q20 and Q30 was 97.06% and 92.45%, respectively. After read mapping analysis, an average of 90.20% of reads were uniquely mapped to the Nipponbare genome sequence (**Supplementary Table 2**). RT-qPCR was used to detect the expression levels of eight identified transcriptome (RNA-ref) differential genes to validate the RNA-ref data. The results of RT-qPCR were positively correlated with those of RNA-ref (**Supplementary Figure 2**).

**Figure 8:**
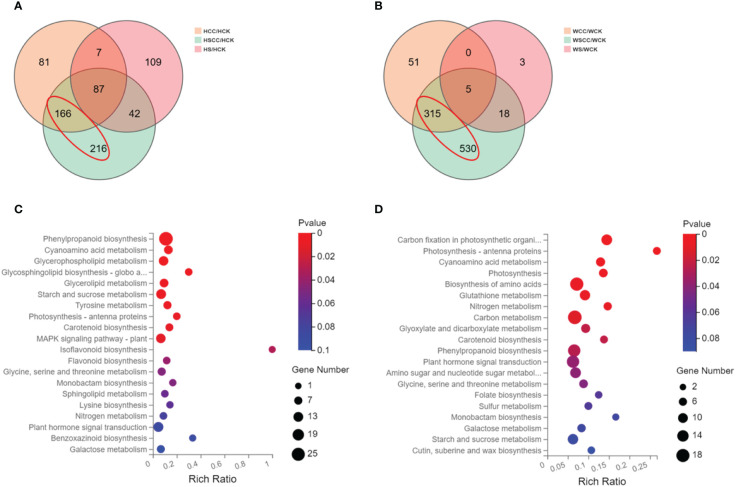
General overview of differentially expressed genes (DEGs). **(A)** Venn graph for the comparisons of HCC vs. HCK, HS vs. HCK, and HSCC vs HCK. **(B)** Venn graph for the comparisons of WCC vs. WCK, WS vs. WCK, and WSCC vs. WCK. **(C)** KEGG enrichment pathway of DEGs in red circles of HHZ. **(D)** KEGG enrichment pathway of DEGs in red circles of WSY.

A total of 3,575 DEGs were identified by pairwise comparison of all the groups. log_2_ (fold change (FC)) and *p < 0.05* thresholds were applied as the standard for identifying differentially expressed genes, and statistical methods were used to analyze the differentially expressed genes under different treatments. In the salt-sensitive HHZ, there were 109, 216 and 81 DEGs only found during the comparison of HS/HCK, HCC/HCK, and HSCC/HCK (**Figure 8A**). And there were 3, 51, and 530 DEGs only found during the comparison of WS/WCK, WCC/WCK, and WSCC/WCK in the salt-insensitive variety WSY (**Figure 8B**). Under salt stress, there were fewer differentially expressed genes in HHZ than in WSY, but there were more differentially expressed genes regulated by CC under salt stress. Both varieties showed that CC regulation under salt stress could change gene expression more than CC regulation.

To investigate the genes encoding proteins directly involved in the metabolic pathways contributing to rice’s CC-mediated salt stress response, we performed a KEGG pathway enrichment analysis of the DEGs. Compared with HS/HCK, the differentially expressed genes under HSCC/HCK were mainly enriched in the Phenylpropanoid biosynthesis (ko00940), Cyanoamino acid metabolism (ko00460), Glycerophospholipid metabolism (ko00564), Glycosphingolipid biosynthesis-globo and isoglobo series (ko00603), Glycerolipid metabolism (ko00561), Starch and sucrose metabolism (ko00500), Tyrosine metabolism (ko00350), Photosynthesis-antenna proteins(ko (ko00196), Carotenoid biosynthesis (ko00906), MAPK signaling pathway-plant (ko04016), Isoflavonoid biosynthesis (ko00943), Flavonoid biosynthesis (ko00941), Glycine, serine and threonine metabolism (ko00260)and Monobactam biosynthesis (ko00261) pathways, in which a gene encoding a WRKY transcription factor was significantly up-regulated (**Figure 8C**). Compared with WS/WCK, the differentially expressed genes under WSCC/WCK were mainly enriched in the Carbon fixation in photosynthetic organisms (ko00710), Photosynthesis-antenna proteins (ko00196), Cyanoamino acid metabolism (ko00460), Photosynthesis (ko00195), Biosynthesis of amino acids (ko01230), Glutathione metabolism (ko00480), Nitrogen metabolism (ko00910), Carbon metabolism (ko01200), Glyoxylate and dicarboxylate metabolism (ko00630), Carotenoid biosynthesis (ko00906), Phenylpropanoid biosynthesis (ko00940), Plant hormone signal transduction (ko04075), Amino sugar and nucleotide sugar metabolism (ko00520) and Glycine, serine and threonine metabolism (ko00260) pathways, in which the gene encoding the WRKY and bZIP transcription factor was significantly up-regulated (**Figure 8D**).

The KEGG enrichment analysis showed that the differentially regulated CC genes of the two rice varieties under salt stress were mainly related to photosynthetic metabolism, such as Carbon fixation in photosynthetic organisms, Photosynthesis-antenna proteins, Photosynthesis, Carotenoid biosynthesis Starch and sucrose metabolism.

**Figure 9:**
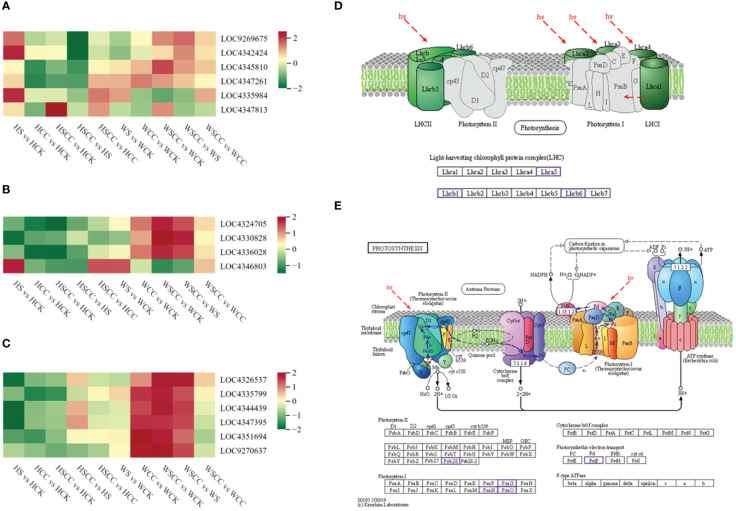
KEGG pathway associated with photosynthesis. **(A)** Heatmap of log_2_FC values of the DEGs enriched in carotenoid biosynthesis (ko00906). **(B)** Heatmap of the DEGs enriched in photosynthesis–antenna proteins (Ko00196). **(C)** Heatmap of the DEGs enriched in photosynthesis (Ko00195). **(D)** KEGG pathway map of the DEGs enriched in photosynthesis – antenna proteins (Ko00196). **(E)** KEGG pathway map of the DEGs enriched in photosynthesis (Ko00195). The KEGG pathway maps with the purple boxes representing the enriched DEGs.

The expression of DEGs enriched in the carotenoid biosynthesis pathway was up-regulated under salt stress and down-regulated after CC treatment. This trend was obvious in the salt-sensitive variety HHZ, especially in the application of CC under salt stress; the DEGs of carotenoid biosynthesis were significantly down-regulated compared with salt stress (**Figure 9A**). The study focused on genes associated with photosynthesis and antenna proteins in the enriched KEGG. Four DEGs were enriched in the photosynthesis-antenna protein pathway. Compared with the control, these DEGs showed down-regulation under salt stress, and up-regulation of CC under salt stress compared with salt treatment alone. Interestingly, the chlorophyll a-b binding protein 1 (*OsCAB1R*, LOC4346803, Os09g0346500) was up-regulated in salt stress compared to CK (**Figures 9B, D**). The upregulation of Chlorophyll a/b binding protein under salt stress was previously reported by Liu et al. to reduce ROS production and enhance photosynthesis, thus leading to salt stress resistance (Liu et al., 2019). There were six DEGs enriched in photosynthesis. Photosystem II reaction center PSB28 protein (*OsPSB28*, LOC4326537, Os01g0938100), photosystem I subunit O (*OsPsaO*, LOC4335799, Os04g0414700), ferredoxin 1 (*OsFd1*, LOC4344439, Os08g0104600), photosystem I reaction center subunit V (*OsPSAG*, LOC4347395, Os09g0481200), photosystem I reaction center subunit N (*OspsaN*, LOC4351694, Os12g0189400) and ferredoxin (*OsFd5*, LOC9270637, Os01g0860601) were down-regulated in salt treated seedlings as compared to CK. The application of CC can up-regulate the DGEs enriched in photosynthesis to alleviate the effect of salt stress, which is more obvious in the salt-tolerant variety WSY (**Figures 9C, E**).

**Figure 10:**
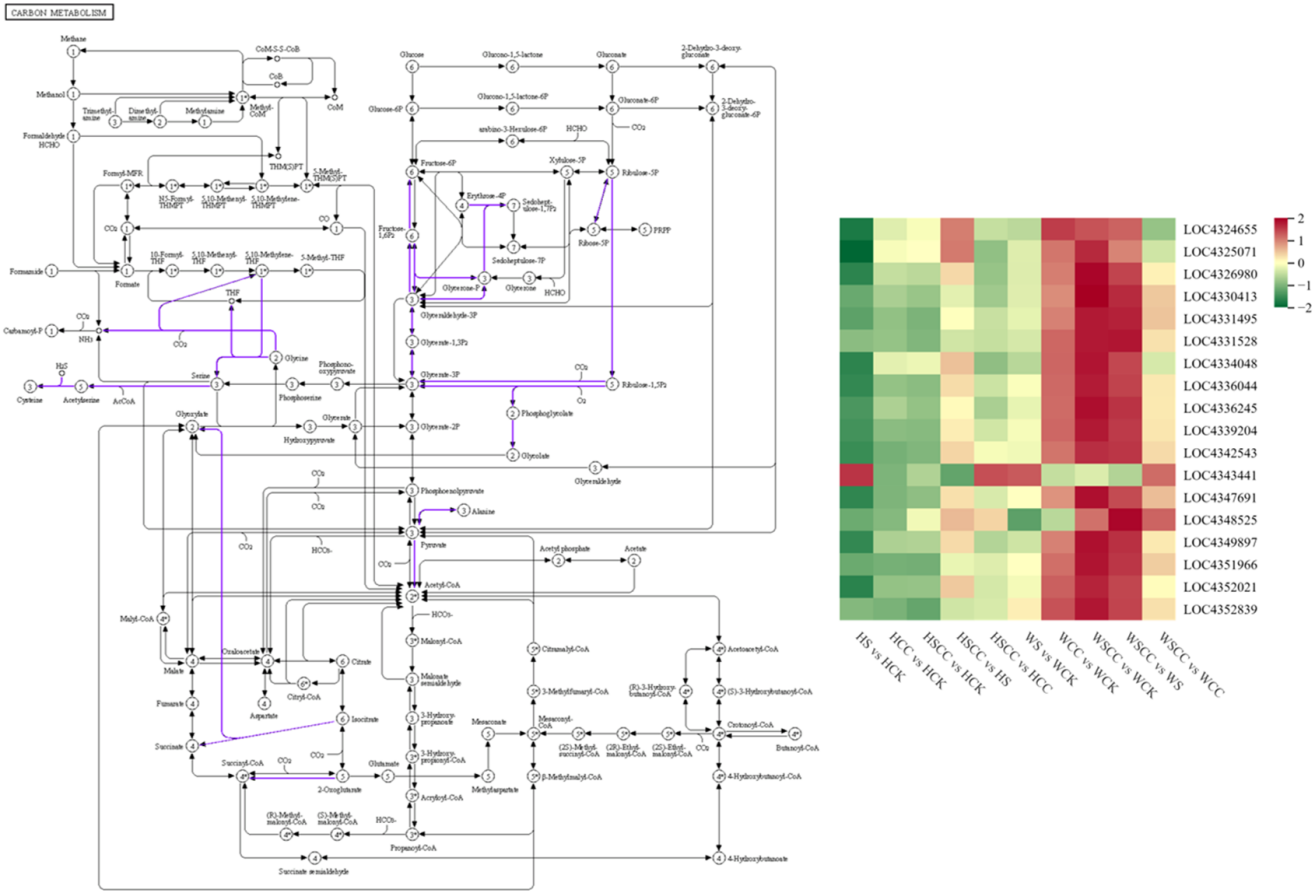
Heatmaps of the log_2_FC values of the DEGs related to carbon metabolism (ko01200). The KEGG pathway maps with the purple boxes representing the enriched DEGs. The maps were developed in the KEGG pathway database using the KEGG pathway mapper by searching Ko IDs of the DEGs on respective pathways.

CC treatment improves rice seedling tolerance against salt stress by increasing the expressions of photosynthesis-antenna proteins and photosynthesis pathway-related genes. Meanwhile, the expression of genes related to carotenoid biosynthesis decreased. Carbon metabolism is closely related to photosynthesis. We analyzed 18 related genes enriched by metabolism. The expression of most differential genes was down-regulated under salt stress compared with CK. After spraying CC under salt stress, these genes were significantly up-regulated to enhance the adaptation of rice seedlings to salt stress. The effects of genes related to regulating carbon metabolism in CC treatment were significant in salt-tolerant variety WSY. Interestingly, compared with controls, isocitrate lyase-like (*OsICL*, LOC4343441, Os07g0529000) was upregulated under salt stress, contrary to the regulatory trend of other related genes enriched in the carbon metabolic pathway (**Figure 10**). *OsICL* has been reported that this gene may be regulated by senescence inducible factors during rice leaf senescence (Lee et al., 2001).

### 3.8 Metabolomic effects of exogenous choline chloride on rice seedlings under salt stress

Metabolite analysis of rice samples was performed to evaluate the effects of CC treatment on the overall metabolism of rice seedling leaves under salt stress. Principal component analysis showed that the metabolites clustered tightly in both positive ion (PC1 = 38.06%, PC2 = 15.45%) and negative ion (PC1 = 58.54%, PC2 = 9.99%) modes, indicating that the samples were reproducible and could be used for further metabolomic analysis (**Supplementary Figure 3**). A total of 931 metabolites were identified in the positive and negative ion modes, with 565 in the positive ion mode and 366 in the negative ion mode. Analyzing the signaling pathways of differentially accumulated metabolites can reveal the main biochemical and signal transduction pathways involved in the differential accumulation of those metabolites. To identify the major pathways that respond to CC in the leaves of rice seedlings under salt stress, we mapped the differentially expressed metabolites to the KEGG pathway and obtained the results of metabolic pathway enrichment.

**Figure 11:**
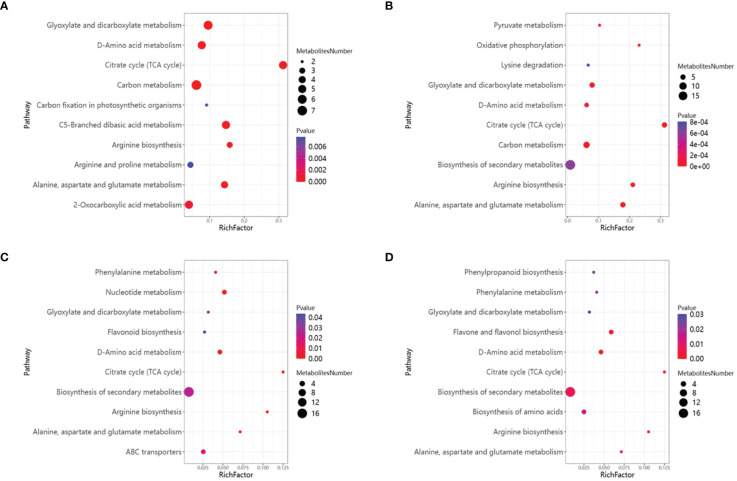
KEGG enrichment analysis of DAMs and the top 10 KEGG annotated scatter plots were sorted according to enrichment factors. **(A)**HCC vs HCK. **(B)** HSCC vs HCC. **(C)** WCC vs WCK. **(D)** WSCC vs WCC.

Between HCC vs HCK, 181 differential metabolites were detected, with 87 up-regulated and 94 down-regulated, were assigned to KEGG pathways, including Glyoxylate and dicarboxylate metabolism (map00630), D-Amino acid metabolism (map00470), Citrate cycle (TCA cycle) (map00020), Carbon metabolism (map01200), Carbon fixation in photosynthetic organisms (map00710), C5-Branched dibasic acid metabolism(map00660), Arginine biosynthesis (map00220) (**Figure 11A**). Between HSCC vs HS, 75 differential metabolites were detected, with 42up-regulated and 33 down-regulated, were assigned to KEGG pathways, including Pyruvate metabolism (map00620), Oxidative phosphorylation (map00190), Lysine degradation (map00310), Glyoxylate and dicarboxylate metabolism (map00630), D-Amino acid metabolism (map00470), Citrate cycle (TCA cycle) (map00020), Carbon metabolism (map01200), Biosynthesis of secondary metabolites (map01110), Arginine biosynthesis (map00220), Alanine, aspartate and glutamate metabolism (map00250) (**Figure 11B**).

Between WCC vs WCK, 107 differential metabolites were detected, with 45 up-regulated and 62 down-regulated, were assigned to KEGG pathways, including Phenylalanine metabolism (map00360), Nucleotide metabolism (map00520), Glyoxylate and dicarboxylate metabolism (map00630), Flavonoid biosynthesis (map00941), D-Amino acid metabolism (map00470), Citrate cycle (TCA cycle) (map00020), Biosynthesis of secondary metabolites (map01110), Arginine biosynthesis (map00220), Alanine, aspartate and glutamate metabolism (map00250), ABC transporters (map02010) (**Figure 11C**). Between WSCC vs WS, 95 differential metabolites were detected, with 45 up-regulated and 50 down-regulated, were assigned to KEGG pathways, including Phenylpropanoid biosynthesis (map00940), Phenylalanine metabolism (map00360), Glyoxylate and dicarboxylate metabolism (map00630), Flavone and flavonol biosynthesis (map00944), D-Amino acid metabolism(map00470), Citrate cycle (TCA cycle) (map00020), Biosynthesis of secondary metabolites (map01110), Biosynthesis of amino acids (map01230), Arginine biosynthesis (map00220), Alanine, aspartate and qlutamate metabolism (map00250) (**Figure 11D**). The DAMs in the four comparison groups were enriched into the TCA cycle. This means that applying CC increases the metabolites produced by rice seedlings involved in the TCA cycle.

### 3.9 Integrative analysis between the transcriptome and metabolome

To understand the relationship between DEGs and DAMs, we conducted an integrative analysis between the transcriptome and metabolome. In association analysis, we analyzed the KEGG pathway shared by DEGs and DAMs in HCC vs HCK, HSCC vs HS, WCC vs WCK, WSCC vs WS (**Supplementary Table 3**). To find out which DEGs and DAMs are related to each other, we plot different omics loadings plots for the variables in the joint part. loading value indicates the explanatory ability of the variable (DEGs/DAMs) in each component (the contribution to the difference between groups), and the positive and negative loading value indicates positive or negative correlation with another group of studies. The greater the absolute value of the load, the stronger the correlation (**Supplementary Figure 4**). According to the loading value results of elements, we screened out the top 25 genes and metabolites with the squared loading value of the first two dimensions for integrated loading map, showing the genes and metabolites with the greatest correlation (**Supplementary Figure 5**). Because the number of DEGs is generally large, it is not conducive to display the association characteristics visually, so all differential genes are screened first. The correlation between the top 250 DEGs and DAMs was shown by heat map (**Supplementary Figure 6**). The DEGs or DAMs in important association positions were shown through correlation network diagram. The data of the top 250 DEGs and DAMs with absolute correlation coefficients greater than 0.5 were selected to draw network diagram (**Supplementary Figure 7**). Taken together, the correlation analysis revealed that specific genes were highly correlated with the products of pathways associated with carbon metabolism, suggesting that these synthetic genes play a crucial role in CC induced carbohydrate synthesis under salt stress.

## 4 Discussion

Salt stress is one of the major abiotic stresses that limit crop growth and productivity worldwide (Kumar et al., 2024). Rice is sensitive to salt stress at the seedling stage (Mishra et al., 2021). Recent studies have elucidated salt stress signaling pathways, including stress response gene activation, ion homeostasis, and growth regulation, as well as other downstream signal-induced responses (Liu et al., 2024; Ma et al., 2024; Zeng et al., 2024).

This study investigated that foliar application of CC enhanced the morphological parameters such as root traits, seedlings height, seedling strength index, seedling fullness, leaf area under salt stress in both rice varieties. These results suggest that CC might promoted the ability of rice seedlings to resist abiotic stress by regulating gene expression and physiological metabolism in plants. In addition, the increase in root-shoot ratio, root length, root surface area, root volume, and root tip number indicated that CC positively affected rice root development, which might be related to CC improving plant adaptability to salt stress. Our work is supported by the findings (Du et al., 2024; Zuo et al., 2024), who reported that application of plant growth regulator improved the morph-physiological traits of rice.

Photosynthesis is crucial biochemical process of energy production in higher plants and improving photosynthetic efficiency is helpful for plants to maintain normal growth and development under salt stress (He et al., 2024). The current findings revealed that most DEGs that respond to CC in rice includes starch and sucrose metabolism, citric acid cycle, carbon sequestration by photosynthetic organs, photosynthetic antenna protein, and carbon metabolism. Salt stress can inhibit photosynthesis by altering enzyme activity (Wang et al., 2024), damaging chloroplasts (Yoo et al., 2019), restricting electron flow (Challabathula et al., 2022), and triggering ROS (Ling et al., 2020). It is essential to enhance overall photosynthesis and crop productivity under salt stress for the survival of all living biota. Foliar application of CC to rice seedlings enhanced chlorophyll content and photosynthetic traits. Similarly, it increased fructose content, starch content and the activity of acid invertase and neutral invertase, indicated that CC might enhance the ability of rice to adapt to salt stress by regulating carbohydrate metabolism. Our results are in agreement with previous studies who observed that exogenous melatonin (Yan et al., 2021a), Hemin (Meng et al., 2023), and gamma-aminobutyric acid (Wang et al., 2023) as plant growth regulators effectively mitigated damage caused by salt stress by improving the photosynthesis of rice.

Abiotic stress inhibit photosynthesis (Feng et al., 2021). The effect of abiotic stress on plant carbohydrates is still controversial. Few studies showed that certain enzymes involved in starch biosynthesis become inactivate under stress, inhibiting the conversion of sucrose to starch and decreasing starch content (Zhang et al., 2024). Other studies showed that early stopping of starch accumulation rather than inhibiting enzymes associated with its biosynthesis is the main cause of reducing starch content (Li et al., 2024). SS decomposes sucrose, regulates starch synthesis and UDPG production. UDPG generates glucose 1-phosphate under the action of UGPase and then ADPG under the action of AGPase, thus synthesizing starch. SPS catalyzes UDPG and fructose 6-phosphate to produce glucose 6-phosphate and UDP. The resulting 6-phosphoglucose is usually rapidly degraded to sucrose by sucrose phosphate phosphatase (SPP). However, SPS and SPP exist in the form of complex in plants, and SPS catalyzes sucrose production, which is irreversible. The decrease in SS activity and the increase in SPS activity led to a decrease in starch synthesis and sucrose accumulation, which might be related to the response of plants to salt stress. These results showed that CC can significantly enhanced photosynthesis, improved carbon metabolism and carbon sequestration pathways to improve rice’s tolerance to salt stress.

Transcriptome and metabolome approaches might further enable us to understand the influence of CC to improve the salt tolerance of rice. Comparative transcriptome analysis showed that both salt stress and CC treatment caused changes in gene expression and SCC treatment had a greater effect on gene expression in rice seedlings. Salt stress adversely affects critical plant physiological processes. Therefore, CC may attenuate the impact of salt on gene expression in rice. Transcriptome and metabolome analysis indicated that CC may enhance plant adaptability to salt stress by regulating the expression of specific genes and altering metabolite accumulation. The differential genes and differential metabolites of CC applied alone and under salt stress were co-enriched into pathways related to photosynthesis and carbohydrate anabolic metabolism. The analysis of transcriptional metabolism is consistent with the physiological traits.

Comparing salt-tolerant varieties and salt-sensitive varieties of rice seedlings, we found differences in the effects of CC on both varieties. WSY (salt-tolerant) rice variety showed stronger growth advantage and metabolic regulation ability under CC treatment, which might be related to its genetic background. Under salt stress the foliar application of CC further improved salt tolerant capability of WSY variety.

The current findings focused on the effect of CC on the seedling stage of rice, but the impact on the mature stage of rice is still unclear. Future studies needs to explore the impact of CC on the salt tolerance of rice at different growth stages and its molecular mechanism. In addition, the amount of CC is small, efficient, low cost, and non-toxic, which can be considered for verification under field conditions to evaluate the potential application of CC in actual agricultural production.

## 5 Conclusions

Foliar application of CC is an effective method that can significantly improve rice seedlings growth and enhance salt tolerance. Exogenous CC can improve the salt tolerance of rice by regulating signal transduction, light trapping process, carbon metabolism, and carbon fixation pathway of photosystem II. These findings provide support for the commercial application of CC in rice cultivation and empirical data and theoretical support for subsequent studies on the relationship between CC treatment and plant stress resistance. It gives a new insight and method for salt-tolerant rice breeding and saline-alkali land improvement. Future studies should explore these regulatory mechanisms to better understand and apply these findings.

## Data availability statement

Publicly available datasets were analyzed in this study. This data can be found here: The datasets presented in this study can be found in online repositories. The names of the repository/repositories and accession number(s) can be found below: The raw sequence data have been deposited in the Genome Sequence Archive in National Genomics Data Center, Beijing Institute of Genomics, Chinese Academy of Sciences, under accession number CRA026838 that are publicly accessible at https://bigd.big.ac.cn/gsa. The metabolite data have been deposited in the National Genomics Data Center, Beijing Institute of Genomics, Chinese Academy of Sciences, under accession number OMIX006512 that are publicly accessible at: https://bigd.big.ac.cn/omix.

## Author contributions

JH: Data curation, Methodology, Project administration, Writing – original draft, Writing – review & editing. MY: Data curation, Writing – original draft. NF: Funding acquisition, Writing – review & editing. DZ: Funding acquisition, Writing – review & editing, Resources. RZ: Resources, Writing – review & editing. YX: Writing – original draft, Writing – review & editing. AK: Writing – review & editing. HZ: Data curation, Writing – review & editing. FM: Investigation, Writing – review & editing. WM: Investigation, Writing – original draft. XD: Data curation, Writing – original draft. XS: Funding acquisition, Project administration, Writing – review & editing. LZ: Project administration, Writing – review & editing.

## Footnotes

1
https://www.kegg.jp/
