# Supplementary material for: Integrated transcriptome and metabolome analysis of salinity tolerance in response to foliar application of choline chloride in rice (Oryza sativa L.)
Source: Front Plant Sci. 2024 Aug 1;15:1440663. doi: 10.3389/fpls.2024.1440663 (PMC11324541; doi:10.3389/fpls.2024.1440663)
Supplement: Supplementary file 4 [file Presentation_1.pptx]

## Slide 1
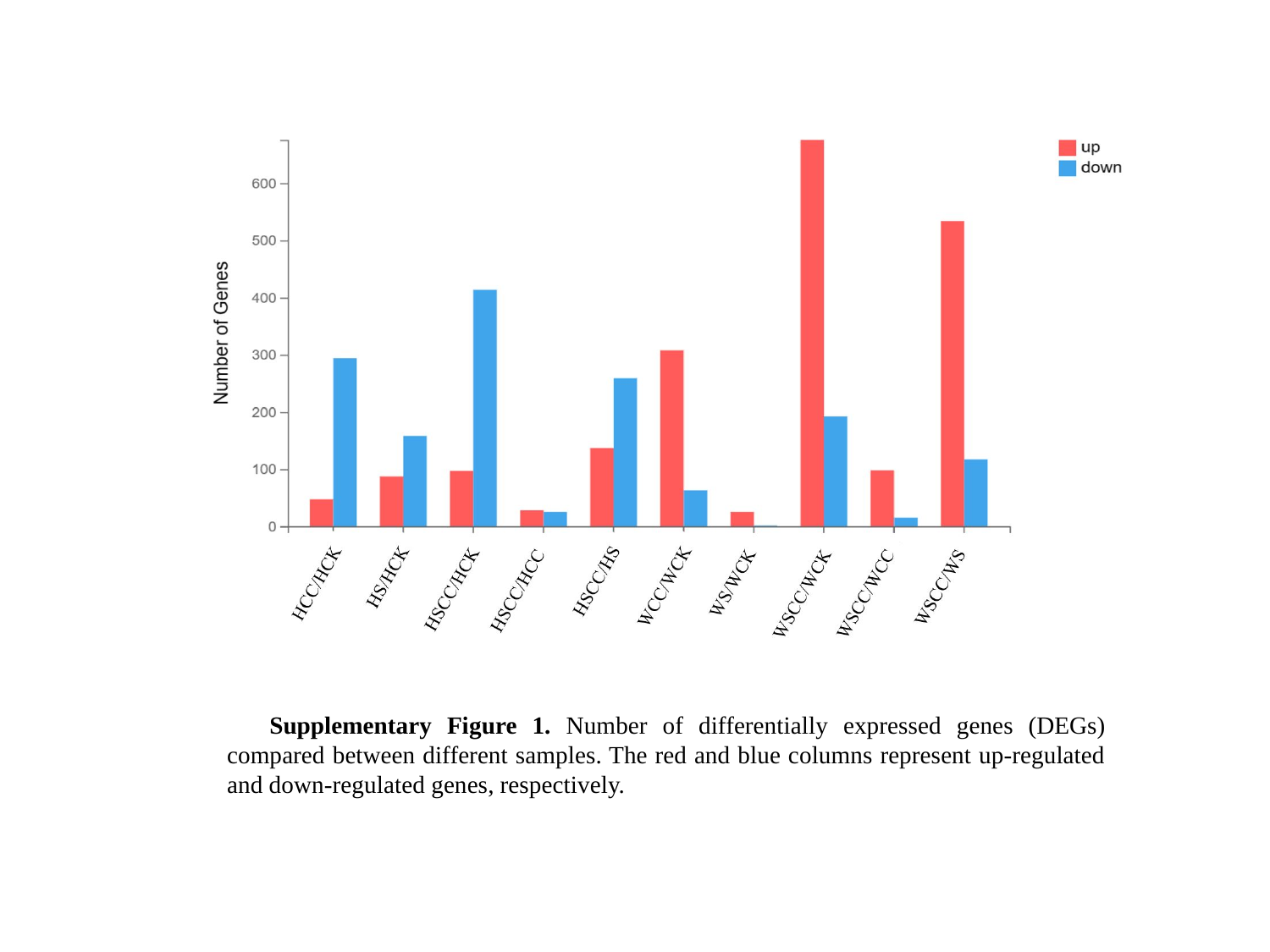

Supplementary Figure 1. Number of differentially expressed genes (DEGs) compared between different samples. The red and blue columns represent up-regulated and down-regulated genes, respectively.
