# Supplementary material for: Integrated transcriptome and metabolome analysis of salinity tolerance in response to foliar application of choline chloride in rice (Oryza sativa L.)
Source: Front Plant Sci. 2024 Aug 1;15:1440663. doi: 10.3389/fpls.2024.1440663 (PMC11324541; doi:10.3389/fpls.2024.1440663)
Supplement: Supplementary file 5 [file Presentation_2.pptx]

## Slide 1
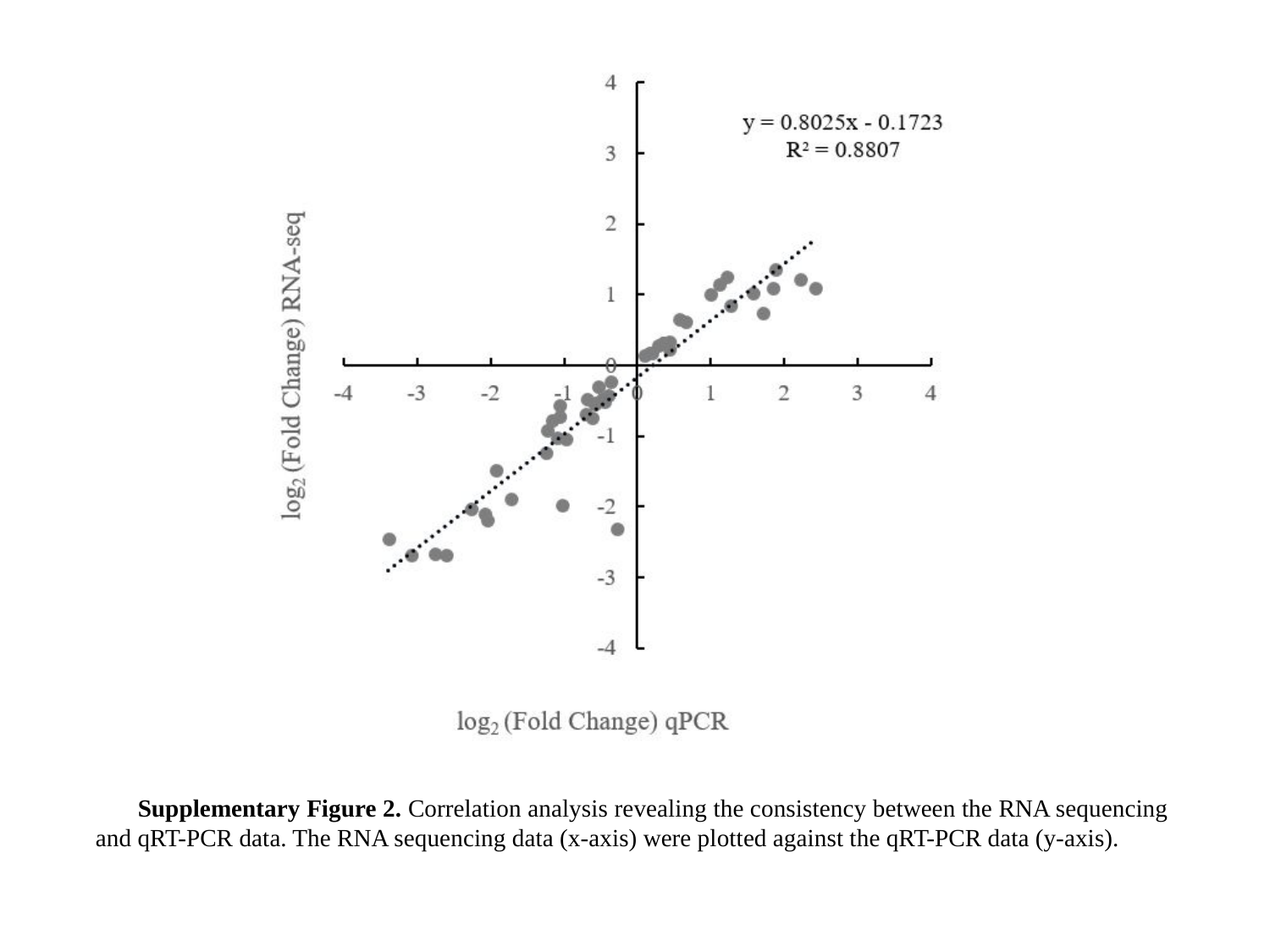

Supplementary Figure 2. Correlation analysis revealing the consistency between the RNA sequencing and qRT-PCR data. The RNA sequencing data (x-axis) were plotted against the qRT-PCR data (y-axis).
