# Supplementary material for: Integrated transcriptome and metabolome analysis of salinity tolerance in response to foliar application of choline chloride in rice (Oryza sativa L.)
Source: Front Plant Sci. 2024 Aug 1;15:1440663. doi: 10.3389/fpls.2024.1440663 (PMC11324541; doi:10.3389/fpls.2024.1440663)
Supplement: Supplementary file 6 [file Presentation_3.pptx]

## Slide 1
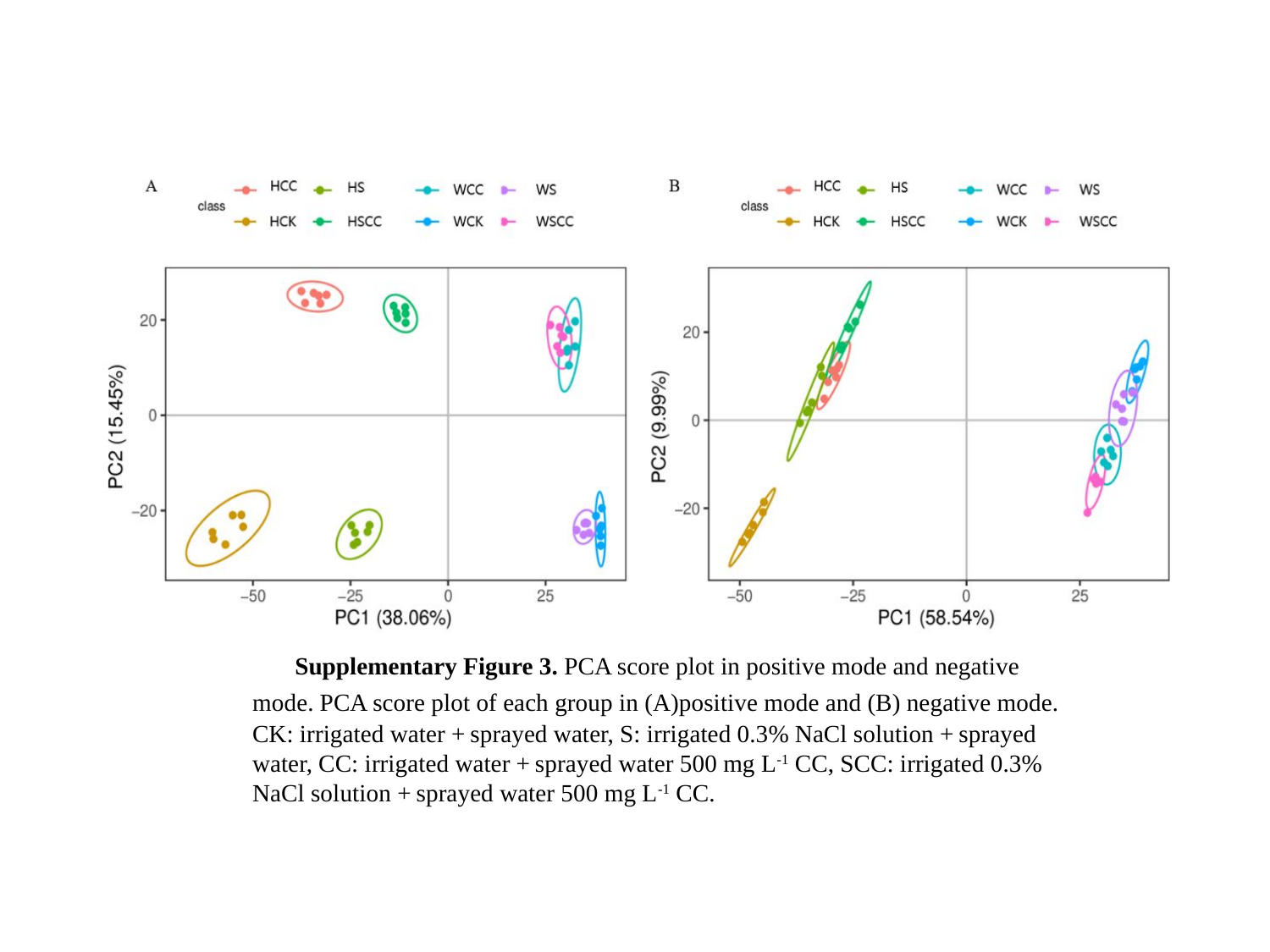

Supplementary Figure 3. PCA score plot in positive mode and negative mode. PCA score plot of each group in (A)positive mode and (B) negative mode. CK: irrigated water + sprayed water, S: irrigated 0.3% NaCl solution + sprayed water, CC: irrigated water + sprayed water 500 mg L-1 CC, SCC: irrigated 0.3% NaCl solution + sprayed water 500 mg L-1 CC.
