# Supplementary material for: Integrated transcriptome and metabolome analysis of salinity tolerance in response to foliar application of choline chloride in rice (Oryza sativa L.)
Source: Front Plant Sci. 2024 Aug 1;15:1440663. doi: 10.3389/fpls.2024.1440663 (PMC11324541; doi:10.3389/fpls.2024.1440663)
Supplement: Supplementary file 7 [file Presentation_4.pptx]

## Slide 1
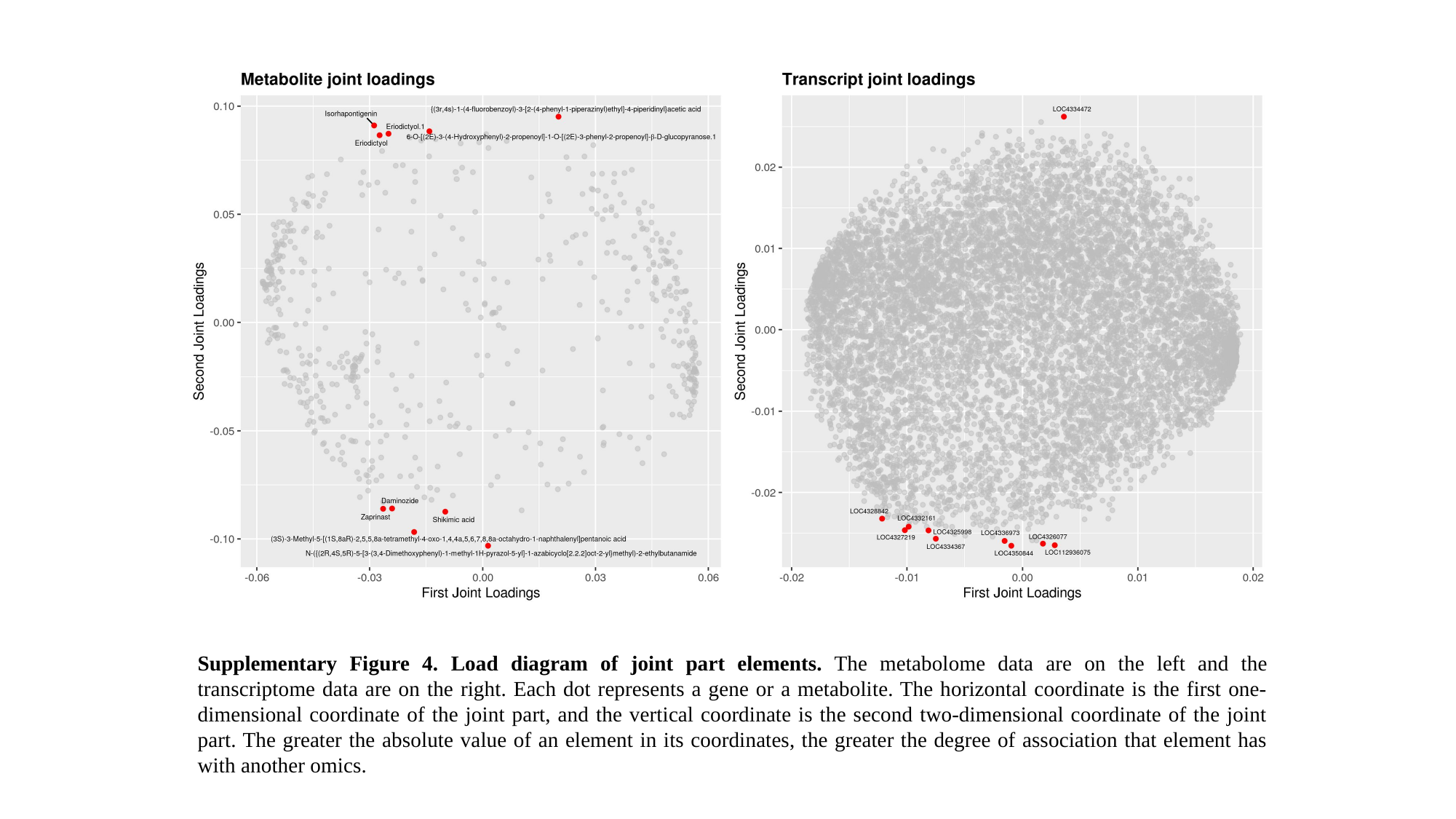

Supplementary Figure 4. Load diagram of joint part elements. The metabolome data are on the left and the transcriptome data are on the right. Each dot represents a gene or a metabolite. The horizontal coordinate is the first one-dimensional coordinate of the joint part, and the vertical coordinate is the second two-dimensional coordinate of the joint part. The greater the absolute value of an element in its coordinates, the greater the degree of association that element has with another omics.
