# Supplementary material for: Integrated transcriptome and metabolome analysis of salinity tolerance in response to foliar application of choline chloride in rice (Oryza sativa L.)
Source: Front Plant Sci. 2024 Aug 1;15:1440663. doi: 10.3389/fpls.2024.1440663 (PMC11324541; doi:10.3389/fpls.2024.1440663)
Supplement: Supplementary file 9 [file Presentation_6.pptx]

## Slide 1
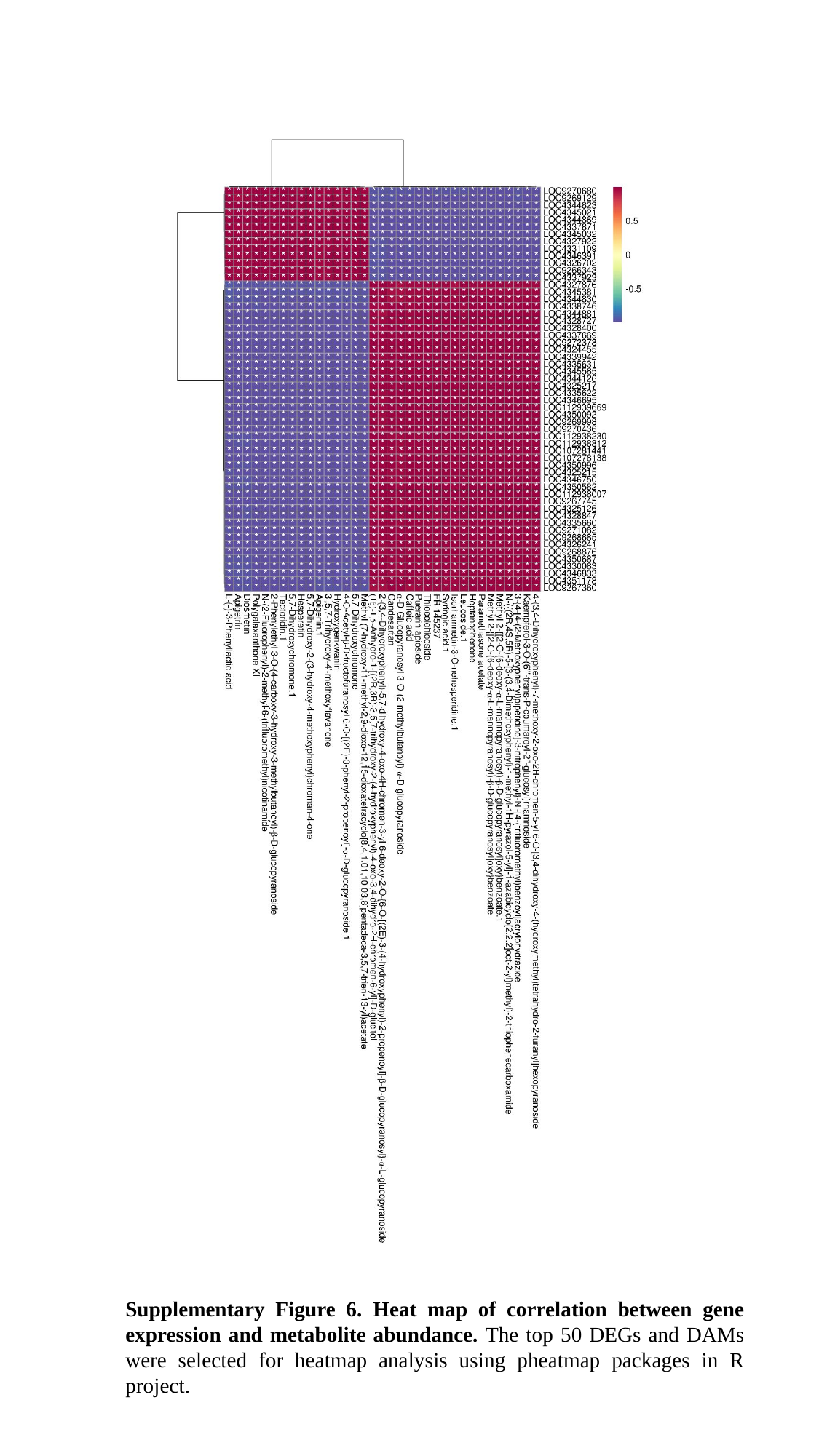

Supplementary Figure 6. Heat map of correlation between gene expression and metabolite abundance. The top 50 DEGs and DAMs were selected for heatmap analysis using pheatmap packages in R project.
