# Supplementary material for: Integrated transcriptome and metabolome analysis of salinity tolerance in response to foliar application of choline chloride in rice (Oryza sativa L.)
Source: Front Plant Sci. 2024 Aug 1;15:1440663. doi: 10.3389/fpls.2024.1440663 (PMC11324541; doi:10.3389/fpls.2024.1440663)
Supplement: Supplementary file 10 [file Presentation_7.pptx]

## Slide 1
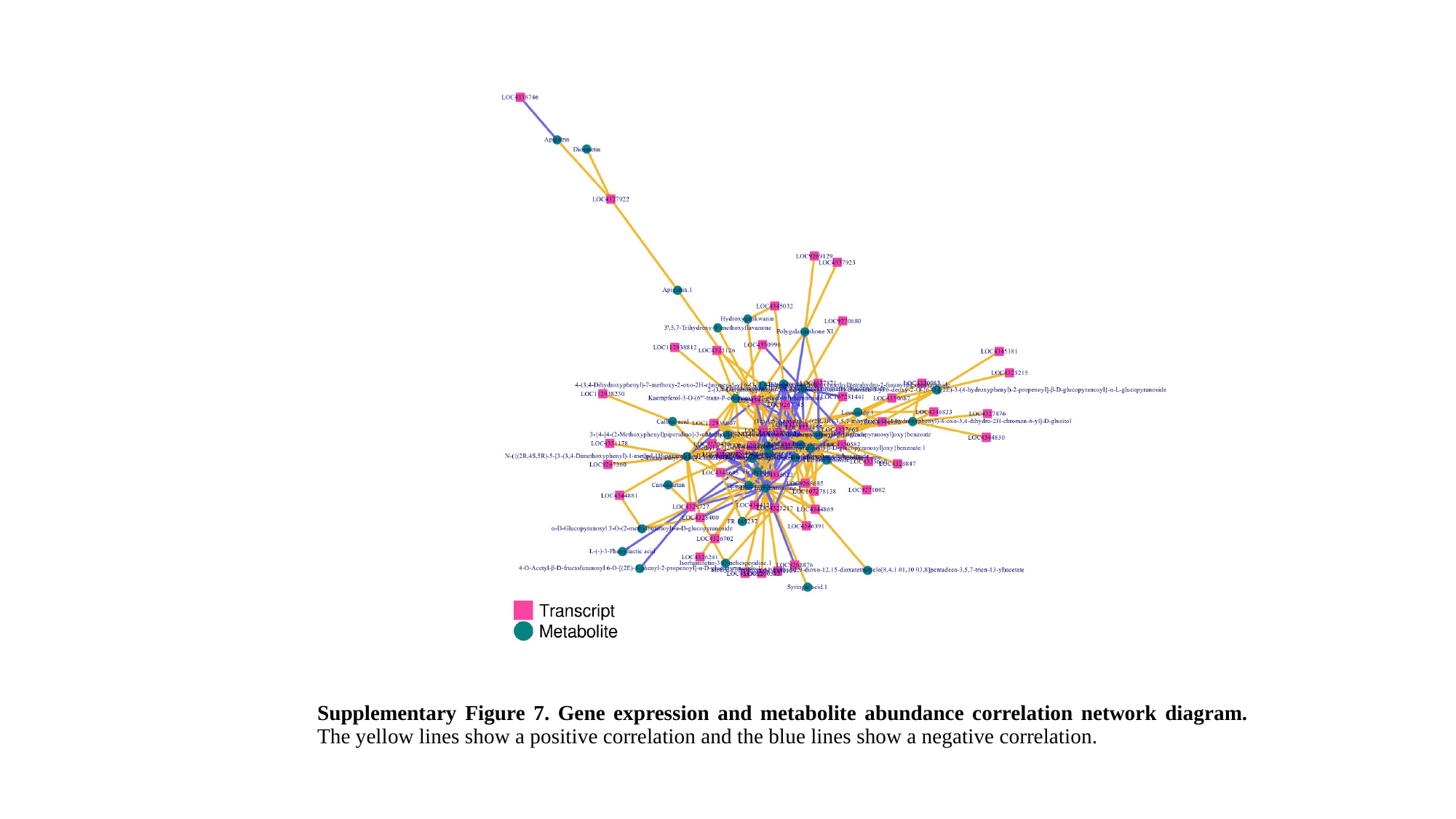

Supplementary Figure 7. Gene expression and metabolite abundance correlation network diagram. The yellow lines show a positive correlation and the blue lines show a negative correlation.
